# Evaluation of Stress Distribution in “All-on-Four” Prostheses: A Three-Dimensional Finite Element Analysis

**DOI:** 10.3390/life16071128

**Published:** 2026-07-07

**Authors:** Eduardo Francisco de Souza Faco, Andressa Paschoal Amoroso, Flávia Priscila Pereira, Luana Ferreira Oliveira, Leandro Lécio de Lima Sousa, André Luis da Silva Fabris, Idelmo Rangel Garcia Junior, José Vitor Quinelli Mazaro, Osvaldo Magro Filho

**Affiliations:** 1 Department of Diagnosis and Surgery, School of Dentistry, São Paulo State University (UNESP), Araçatuba 16010-380, SP, Brazil; eduardo.faco@unesp.br (E.F.d.S.F.); andre.fabris@hotmail.com (A.L.d.S.F.); irangel@unesp.br (I.R.G.J.); 2 Department of Dental Materials and Prosthodontics, School of Dentistry, São Paulo State University (UNESP), Araçatuba 16010-380, SP, Brazil; andressapamoroso@gmail.com (A.P.A.); jose.mazaro@unesp.br (J.V.Q.M.); 3 UNIFUNEC—Integrated Colleges of Santa Fé do Sul, Santa Fé do Sul 15775-000, SP, Brazil; flaviappereira@hotmail.com; 4 Department of Periodontics, Universus Veritas University Guarulhos—UNG, Guarulhos 07023-070, SP, Brazil; odontologialeandrosousa@gmail.com

**Keywords:** dental implants, biomechanics, finite element analysis

## Abstract

The growing demand for implant-supported rehabilitative prosthetic treatments has reinforced the need to optimize biomechanical performance, particularly regarding force distribution. This study aimed to evaluate the stress distribution generated by different configurations of full-arch implant-supported prostheses using three-dimensional finite element analysis. Two mandibular models were created using SolidWorks 2010 (SolidWorks Corp., Waltham, MA, USA) and Rhinoceros^®^ 3D 4.0 (NURBS Modeling for Windows, USA). Each model represented a mandible restored with a full-arch fixed prosthesis supported by external hex implants (4.0 × 13.0 mm; Master, Conexão Sistemas de Prótese, São Paulo, Brazil) placed in the interforaminal region, differing only in implant angulation. Model 1 included four implants positioned perpendicular to the alveolar ridge, whereas Model 2 represented the All-on-Four configuration with distal implants tilted at 30°. The prosthesis was modeled in acrylic resin with a NiCr metal framework. The geometries were exported to FEMAP 11.0 for mesh generation. Axial loading of 300 N was applied bilaterally (75 N per tooth), and oblique loading of 150 N was applied unilaterally (75 N per tooth) on the first premolars and first molars. Obtained using NEiNastran^®^ 9.2 showed that the tilted-implant model exhibited higher stress concentrations under both loading conditions. The All-on-Four configuration generated the highest stress levels, particularly around the distal implants. The null hypothesis of this study was that there would be no difference in stress distribution among full-arch implant-supported prostheses supported by straight implants and those rehabilitated according to the All-on-Four concept with tilted distal implants.

## 1. Introduction

The restoration of function in fully edentulous patients using implant-supported prostheses was first proposed by Brånemark et al., leading to significant improvements in patients’ quality of life compared with conventional dentures [1,2,3,4]. One of the main limitations of osseointegrated implant rehabilitation was the reduced bone availability in the posterior regions of atrophic mandibles, particularly beyond the mental foramina, which made rehabilitation challenging [5].

To overcome this issue, the “All-on-Four” concept, described by Maló et al. (2003), was introduced as an enhancement of the immediate loading technique [6]. This approach involves the placement of only four implants in the mandible, with the two posterior implants tilted distally at 30° or 45° [7,8,9]. This configuration results in a lower-cost rehabilitation due to the reduced number of implants and prosthetic components, allows the use of longer distal implants that improve primary stability and reduce cantilever extension, and preserves vital structures such as the inferior alveolar nerve, eliminating the need for nerve lateralization [10].

One ongoing discussion concerns the efficiency of stress distribution in tilted implants. Some studies have suggested that when subjected to occlusal loading, these implants may induce greater stress on the surrounding bone and implant structures; however, this remains controversial [11,12,13]. Despite this, the literature reports favorable success rates for rehabilitation with tilted implants [14,15,16]. Therefore, stress analysis studies are essential to better understand the biomechanical behavior of full-arch prostheses supported by only four implants—two of which are tilted posteriorly—and to evaluate the mechanical influence of the cantiléver [17,18,19].

Currently, the finite element method is an efficient and reliable method for studying stress distribution between interfaces [14,20,21,22,23]. Compared to the traditional photoelasticity method, it offers the advantages of precision and stability. In this context, the methodology allows trend analysis to simulate an “in vivo” reality in computational models, which would not be ethically permitted in comparative clinical trials [24]. Thus, the use of 3D finite elements allows evaluation of stress distribution in implants and peri-implant bone tissue, identifying possible changes in geometry and surface that can benefit bone tissue in terms of predictability [25,26].

Finally, longitudinal studies and clinical follow-ups of patients rehabilitated using the All-on-Four technique have demonstrated high success rates, reinforcing the importance of investigating the biomechanics of this type of rehabilitation [25,27]. Therefore, the aim of this study was to comparatively analyze the stress distribution in the surrounding bone, implants/components, and prosthetic structures in full-arch and All-on-Four configurations using three-dimensional finite element analysis.

The null hypothesis of this study was that there would be no difference in stress distribution among full-arch implant-supported prostheses supported by straight implants and those rehabilitated according to the All-on-Four concept with tilted distal implants.

## 2. Materials and Methods

For this study, two three-dimensional models were made, each representing a mandible with a protocol-type prosthesis with external hexagon implants (4.0 × 13.0 mm) (Conexão Sistemas de Prótese Ltd., São Paulo, Brazil) installed in the interforaminal region, varying the inclination of the implants. The three-dimensional drawings were simulated using the Solidworks 2010 graphic modeling program (SOLIDWORKS Corp., Headquarters. Waltham, MA, USA). Table 1, below, illustrates the specifications of the models that were used in this study.

For the fabrication of the implant, a hexagon (Conexão Sistemas de Prótese Ltd., São Paulo, Brazil) was used as a reference, with the characteristics of each proposed model (Table 1) (Appendix A). The drawings were created in the Solidworks 2010 program, which made it possible to reproduce, with high fidelity, the dimensions of the internal and external shape of the implant, the standard and angled abutments, and the coping.

The 3D design of the mandible was generated in Solidworks 2010 with modeling in Rhinoceros^®^ 3D 4.0 (NURBS Modeling for Windows, USA), and the implants were inserted into the interforaminal region using the same program. For the fabricati19on of the metal framework, a cantilever prosthetic bar made of NiCr alloy was used as a reference, with a length of 15 mm, 6 mm thickness, and 4 mm height in the form of an arch connecting to the coping. Furthermore, the distance between bone and prosthesis corresponds to the gingiva, which is essential for a realistic 3D model, ensuring that the study reflects how the soft mucosa reduces mechanical stress on the implants and bone.

**Figure 1:**
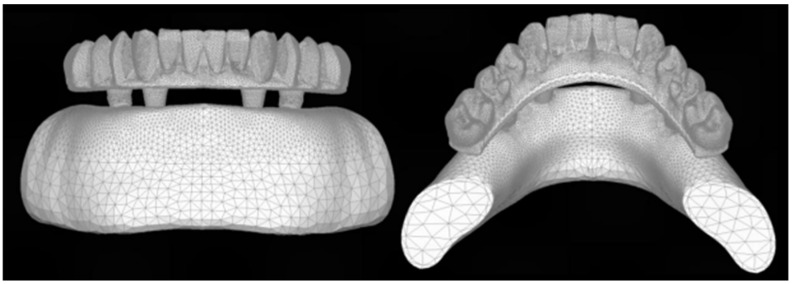
Finite Element Mesh.

For the design of the Protocol type prosthesis, a prosthesis made of acrylic resin was used as a reference, digitized from a Dental Wings 3D scanner (Innbec Technologies Inc., Montreal, QC, Canada) and modeled in the Rhinoceros^®^ 3D 4.0 program. After the models were made, they were exported to the FEMAP 11.0 finite element pre- and post-processing program to create the three-dimensional mesh (Figure 1), into which the mechanical properties of the materials used, obtained from the literature (Table 2), were incorporated. The materials were considered homogeneous, isotropic, and linear.

The contacts between implant/cortical bone, implant/trabecular bone, cortical bone/trabecular bone, and the prosthesis fixation screws were assumed to be bonded, while the contact between structure/implant was assumed to be juxtaposed. Boundary conditions were established as fixed on the three axes (x, y and z) on the lateral surfaces of the cortical and trabecular bone, with the rest of the assembly free of restrictions. The axial load applied to the model was 300 N, bilaterally, divided into 4 points of 75 N, on the first premolars (mesial implants) and first molars (distal implants) in the main groove in its median portion, and an oblique load of 150 N, unilateral (220 N), divided into 2 points of 75 N, on the first premolar (mesial implant) and the first molar (distal implant), on the vestibular face, close to the cusp to simulate mastication, in the nodal force modality [9,28,29].

Next, the simulated mathematical problem was solved using the finite element program NEi Nastran 9.0, and the results were visualized using individual von Mises Stress and Maximum Principal Stress maps for each proposed model, with the aim of verifying the variations in stress distribution in the bone, implant, intermediates, and metal structure.

## 3. Results

**Figure 2:**
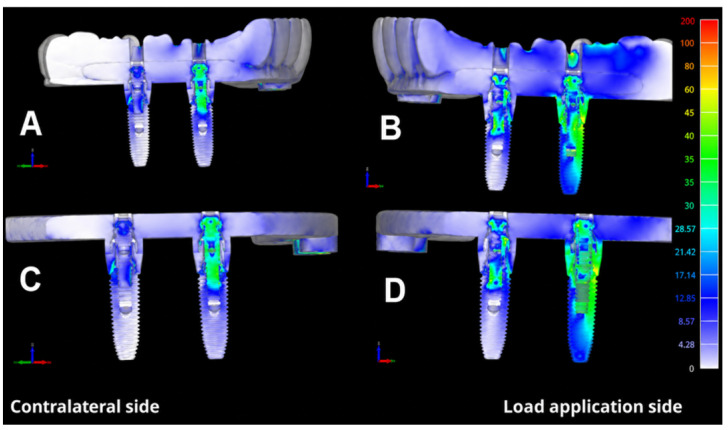
Sagittal section of the prosthesis, framework, and implants. von Mises stress map, contralateral side (**A**,**C**) and load application side (**B**,**D**)—Oblique Load—Straight Implants Model.

In the analysis of von Mises stresses, in the model with straight implants, in a sagittal section showing the implants and prosthesis infrastructure, it was possible to assess that there was a greater concentration of stresses in the load application region of the distal implant in the range of 200 MPa (Figure 2).

**Figure 3:**
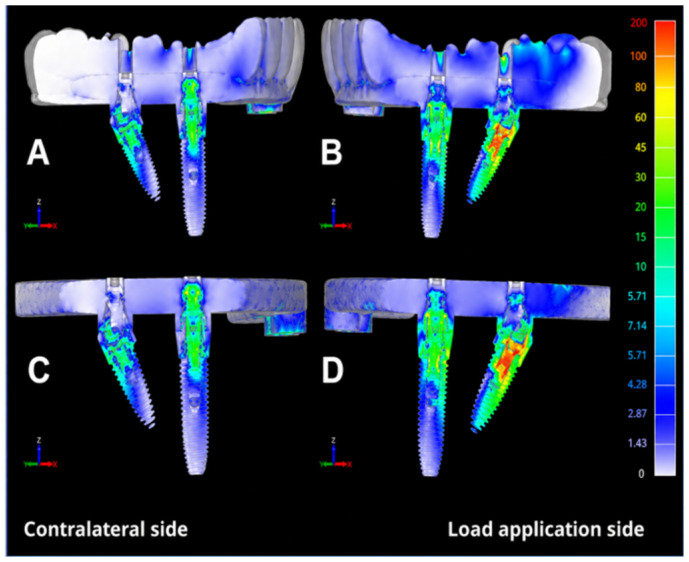
Sagittal section of the prosthesis, framework, and implants. von Mises stress map, contralateral side (**A**,**C**) and load application side (**B**,**D**)—Oblique Load—Angled Implants Model.

In the model with angled implants, a stress range was observed with a maximum value of 200 MPa in the distal implant, with the highest stress concentrations at the contact interfaces of the abutment screw structures and in the distal portion of the abutment seating on the implant platform (200 MPa) and concentrations on the order of 40 MPa from the first implant thread to the median portion (Figure 3).

The structures that exhibited the highest stress concentrations, with the application of loads in the von Mises stress analysis, are illustrated in the table below (Table 3).

**Figure 4:**
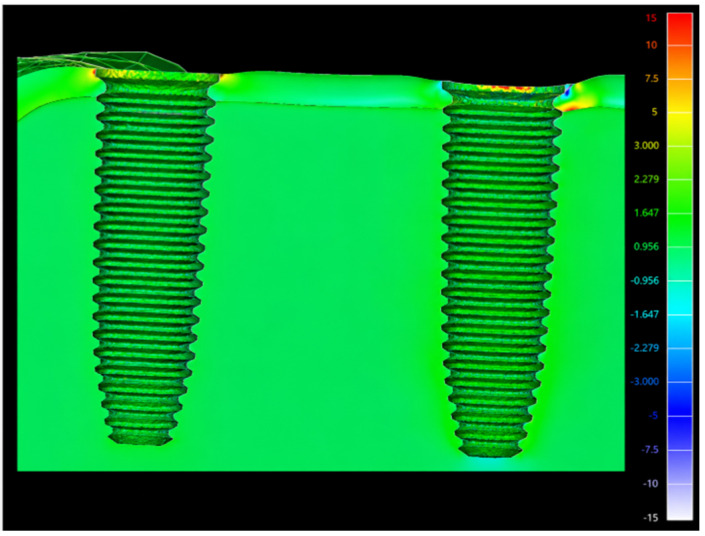
Sagittal section of cortical and trabecular bone—Maximum Principal Stress Map—Axial Load—Straight Implant Model.

In an analysis of the principal maximum stresses in the straight implant model, it was observed that there is a greater concentration of stresses in the interface region of the cortical bone with trabecular bone in both implants, but mainly in the distal implant, with stresses in the range of 15 MPa. In the top view, it is possible to observe that there was a greater concentration of compressive stresses, mainly for the distal implant, in the range of −15 MPa, in its distal portion, as shown in Figure 4. The bottom view shows the greater extent of tensile stresses for the distal implant, on its distal face, in the range of 15 MPa, as shown in Figure 4.

In an enlarged view of the interface between the bone cortex and the distal implant, it was possible to observe that there is a greater area of concentration of tensile (15 MPa) and compressive (−15 MPa) stresses throughout the bone extension in the distal implant, around 7.5 MPa, Figure 4.

**Figure 5:**
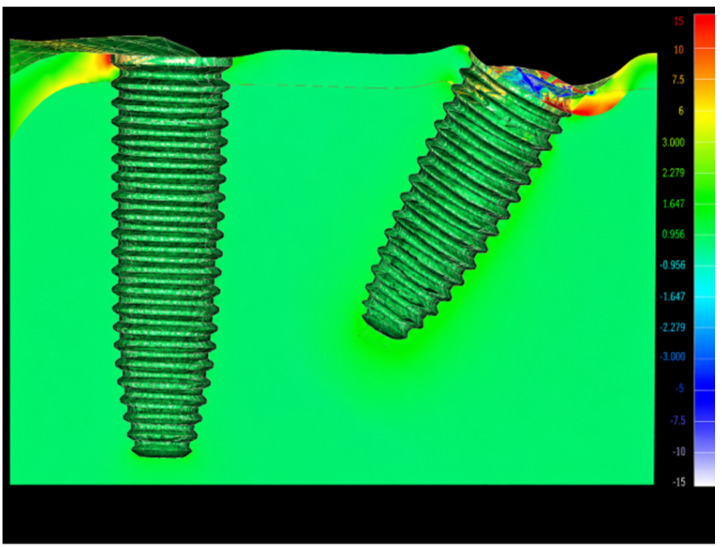
Sagittal section of cortical and trabecular bone—Maximum Principal Stress Map—Axial Load—Inclined Implant Model.

In the angled implant model, it was observed that there is a greater concentration of stresses in the region of the interface between the cortical bone and trabecular bone, and the contact area of this with the first threads of the distal implant on its distal face, with stresses in the range of 15 MPa. Note that in the mesial portion of the cortical bone, the stresses are minimal, in contrast to what occurs in the mesial implant, which shows a maximum tensile stress on the order of 15 MPa on its mesial face, as shown in Figure 5.

Table 4 below shows the maximum peak stress, in the main maximum stress analysis, considering the enlarged view of the bone cortex interface with the implant.

## 4. Discussion

Analyses of maximum principal stress and von Mises stress showed that distally tilted implants following the All-on-Four concept exhibited higher stress concentrations in all simulated conditions when compared with straight implants, consistent with previous literature [9,30,31]. The axial and oblique loads applied sought to simulate masticatory conditions, and the implants were considered osseointegrated [4,7,28,32].

In the von Mises stress maps under bilateral axial loading, the models with tilted implants presented higher stress values concentrated mainly on the abutment screw and at its seating interface with the implant platform. In the model with straight implants, stress values were lower; however, the highest concentration still occurred on the distal implant, particularly on its distal surface, similar to the behavior of the anterior implants. The unilateral oblique load produced a similar stress distribution to axial loading in the tilted-implant model, with greater intensity than in the straight-implant model. However, in both models, on the contralateral side, the mesial implant exhibited the greatest stress. The increased stress concentration on distal implants likely resulted from the cantilever, as described in the literature [23,33,34,35].

Maximum principal stress analysis, which evaluates the implant–bone interface and allows identification of potential sites of physiological bone failure or resorption [36], showed higher stress concentrations in cortical bone than in trabecular bone in all loading conditions for the tilted-implant models, particularly at the distal implant. This greater concentration of cortical stresses has also been reported in studies using the same methodology [8,37,38]. Axial loading generated the highest tensile stresses on the distal aspect of these implants, similar to the oblique loading pattern.

In the present study, higher cortical bone stress concentrations were found in tilted implants and under oblique loading, in agreement with Begg et al. (2009), who used photoelastic analysis and suggested that tilted implants may induce greater peri-implant deformation [37]. Similarly, Naini et al. (2011) found no advantages when comparing tilted and straight implants in All-on-Four systems, since reducing the cantilever decreased stresses around the anterior implants, but tilted implants still received higher stress concentrations, corroborating Takahashi et al. (2010) [9,33].

External hex implants were used because this connection system was originally recommended for the technique; however, this may have contributed to higher stress concentrations. According to De Faria Almeida et al. (2014) [11], external hex implants may show higher stress concentrations compared with Morse taper implants in studies involving single-unit prostheses.)

The model with tilted implants exhibited higher stress concentrations in all situations, both in prosthetic components and in adjacent bone, which reported that tilted implants may lead to mechanical complications such as screw fractures, abutment failures, fracture of prosthetic materials, or framework failure. These results differ from Baggi et al. [16], who demonstrated effective and uniform stress distribution in prostheses supported by tilted implants [6,34,35,39].

The implant inclination used in the present study was 30°, following the All-on-Four protocol described by Maló et al. [6]. However, Malhotra et al. [32] reported higher stress for inclinations of 40° and 45°, indicating that increasing the angulation does not necessarily produce proportional increases in stress [20]. Nevertheless, despite the magnitude of stresses observed in bone tissue, these values did not exceed the physiological limits of resistance, supporting the biomechanical feasibility of the technique [11,20,36,40].

Although the All-on-Four concept resulted in higher stress concentrations in prosthetic structures and adjacent bone, it offers important advantages, such as avoiding anatomical structures like the inferior alveolar nerve, allowing the placement of longer implants. As stated by Takahashi et al. [33,34] reducing the distal cantilever and achieving an effective distribution of implants across the four mandibular regions are additional benefits, also emphasized by Butura et al. (2011) [8]. When performed with adequate implant stability, the technique is supported by the literature, including studies by Francetti et al. (2012) and Francetti et al. (2008) [40,41].

Regarding methodological limitations, they are inherent to computational simulations. However, this approach provides valuable parameters for clinical comparison and biomechanical protocol development [11,36]. In this study, we consider tilted implants a viable option for full-arch rehabilitation, as the stress magnitudes remained within the physiological limits of bone and titanium. Moreover, clinical studies report high success rates for All-on-Four rehabilitations in atrophic mandibles [4,7,8,35,39,41]. A limitation of the present study is that mesh convergence, sensitivity analysis, and experimental validation were not performed. Therefore, the results should be interpreted as a comparative biomechanical assessment between configurations rather than as absolute predictors of clinical performance.

The results demonstrated that the All-on-Four configuration with tilted distal implants generated higher stress concentrations in both prosthetic components and surrounding bone compared with the configuration using straight implants. Therefore, the null hypothesis was rejected.

## 5. Conclusions

Based on the methodology employed, it was possible to conclude that the models with tilted implants exhibited higher stress levels in all analyzed structures under both loading conditions. The abutment screw showed the highest stress concentration among the components. The greatest stress accumulation occurred at the level of the first implant threads in the tilted-implant models, where tensile stresses predominated at the cortical bone–implant interface.

## Figures and Tables

**Table 1:** Models’ specifications.

Model	Descrition
1	Mandibular fixed full-arch prosthesis supported by four externally hexed implants (4.0 × 13.0 mm) placed perpendicular to the alveolar ridge (straight implants).
2	“All-on-Four” prosthesis supported by four externally hexed implants (4.0 × 13.0 mm), with the distal implants tilted 30°.

**Table 2:** Material Properties.

Materials	Elastic Modulus (E) (GPa)	Poisson’s Ratio (μ)	References
Cancellous Bone	1.37	0.30	Sertgoz (1997) [24]
Cortical Bone	13.7	0.30	Sertgoz (1997) [24]
Titanium (implant, abutment)	110.0	0.35	Sertgoz (1997) [24]
Ni-Cr Alloy	206.0	0.33	Anusavice & Hojjatie (1987) [23]
Cancellous Bone	1.37	0.30	Sertgoz (1997) [24]
Cortical Bone	13.7	0.30	Sertgoz (1997) [24]
Titanium (implant, abutment)	110.0	0.35	Sertgoz (1997) [24]
Ni-Cr Alloy	206.0	0.33	Anusavice & Hojjatie (1987) [23]
Cancellous Bone	1.37	0.30	Sertgoz (1997) [24]

**Table 3:** Von Mises Stress—Structures Exhibiting the Highest Stress Level.

Load	Mesial Implant	Distal Implant
**Axial**		
Straight Implants	abutment screw/coping screw	abutment–implant platform
Angled Implants	coping screw/abutment screw/abutment	abutment screw/abutment
**Oblique**		
Straight Implants	abutment screw/coping screw	abutment screw/implant platform
*Contralateral*	abutment screw/coping screw	implant platform
Angled Implants	Abutment	abutment/abutment screw
*Contralateral*	coping screw/abutment screw	implant platform/abutment screw

**Table 4:** Maximum Principal Stress—Peak Stress Value (MPa).

Load	Mesial Implant	Distal Implant
**Axial**		
Straight Implants	7.5	15 to −15
Implants Angled	15	15 to −15
**Oblique**		
Straight Implants	−1.667	10 to −5
*Contralateral*	1.667	−2.778
Implants Angled	10 to −5	15 to −15
*Contralateral*	7.5	−3.889 to 5

## Data Availability

The original contributions presented in the study are included in the article, and further inquiries can be directed to the corresponding authors.

## Abbreviations

FEM	Finite Element Method
MPa	Megapascal
3D	Three-dimensional
N	Newton
mm	Millimeters
NiCr	Nickel–Chromium

## Appendix A

### Detailed Description and Illustrations of the Methodology: Fabrication of the Models

- **Finite Element Method**

The methodology used in this study was the three-dimensional finite element method. Finite element analysis was initially applied in the 1960s to solve structural problems in the aerospace industry. Since then, its applications have expanded to include the resolution of heat transfer, fluid flow, mass transport, and electromagnetic field problems [38].

This method is a technique that mathematically reproduces the behavior of a given physical system; in other words, a physical prototype can be evaluated through the creation of an accurate mathematical model. To achieve this, a model with a geometric representation of the actual physical structure under investigation is required [42]. This representation is constructed by dividing the body into a discrete number of elements through a procedure known as discretization. These elements are interconnected by points called “nodes,” forming a network or mesh. The stresses generated in this model after fixation, when an external load is applied, are calculated for each element. The integration of the behavior of all individual elements yields the structural response to the external stimulus, resulting in the identification of stress distribution areas [38,42,43].

- **Software Used**

*SolidWorks*

SolidWorks is a 3D parametric solid modeler that enables the construction of highly realistic three-dimensional models. It offers a wide range of tools for developing parts and assemblies, as well as various functionalities for working with sheet metal, molds, metal structures, and surfaces [44,45]

*Rhinoceros*

Rhinoceros 3D is a three-dimensional modeling software that constructs solid models from complex surfaces. One of its most notable features is its extensive set of import and export options. The large number of supported file formats allows Rhinoceros to function as a “converter,” bridging compatibility gaps between different software used during project development.

*InVesalius*

InVesalius (version 3.1.1) is a healthcare-oriented software that reconstructs three-dimensional virtual models (3D) from two-dimensional (2D) images obtained via computed tomography or magnetic resonance imaging.

- **Model Fabrication**

*Protocol Prosthesis*

A mandibular acrylic resin Protocol Prosthesis was obtained using an experimental dental mannequin.

The prosthesis was digitized using a Dental Wings 3D scanner (Innobec Technologies Inc., Montréal, QC, Canada). After scanning, the 3D model was generated using Rhinoceros^®^ 3D 4.0 for structural modeling, with additional refinements performed in SolidWorks^®^ 2006. The protocol-type prosthesis was then connected to the implant abutment (standard and angled) and to the coping after its fabrication.

*Implant*

A Master implant (Conexão Sistema de Prótese Ltd., São Paulo, Brazil) with an external hexagon, 10 mm in length and 4.0 mm in diameter, was used as a reference. For simplification of the implant, abutment, and screw models, SolidWorks^®^ 2010 was employed. After simplification, the implant model was exported to Rhinoceros^®^ 4.0 for connection to the protocol prosthesis, and the full assembly was then exported to SolidWorks^®^ 2010 for insertion into the bone structure.

*Trabecular and Cortical Bone*

Trabecular and cortical bone structures were obtained from a reconstructed computed tomography scan of a cross-sectional slice in the molar region. These data were transferred to InVesalius (CTI, São Paulo, Brazil), which generated a three-dimensional mandibular model later imported into Rhinoceros^®^ 4.0 for conversion and editing into a format compatible with the FEMAP 11.0 pre-processor (integrated into NEiNastran^®^ 9.2) for finite element analysis.

*Finite Element Model Development*

After generating the models, they were exported to NEiNastran^®^ 9.2 (Noran Engineering, Inc., Westminster, CA, USA), where geometric modeling was performed. Minor corrections were applied to solve issues that would hinder high-quality mesh generation. Automatic correction tools in FEMAP 11.0 were used to eliminate overlapping points and open surfaces caused by precision errors.

Mechanical properties were then assigned to each material, including Young’s modulus and Poisson’s ratio, based on values reported in the literature [9,43,46,47]. All materials were considered isotropic, linear, and homogeneous [43,48]. Following property assignment, the finite element mesh was generated using 10-node parabolic tetrahedral solid elements. Mesh quality control ensured accurate representation of the physical phenomena under study.

Boundary conditions were defined by establishing contact constraints and loading conditions. The implant was bonded to the cortical and trabecular bone, and all other components were also modeled with bonded contact, preventing penetration, sliding, or separation at the interfaces.

An axial load of 300 N was applied bilaterally in the premolar and first molar regions, and an oblique load of 150 N (at 20°) was applied unilaterally to the buccal surfaces of the first premolar and first molar. After configuring the boundary conditions and confirming that linear analysis was appropriate for the system, the NEiNastran^®^ 9.2 solver was executed. The results were then imported into FEMAP 11.0 for visualization and post-processing of von Mises and maximum principal stress maps.

The analysis was performed on a workstation (HP Z200, Hewlett-Packard Company, Palo Alto, CA, USA).
